# Generation of a senescent synovial organoid model using rat synovial fibroblasts treated with oxidative stress

**DOI:** 10.1186/s41232-026-00436-2

**Published:** 2026-08-05

**Authors:** Sunghyun Lee, Kentaro Endo, Ichiro Sekiya

**Affiliations:** Center for Stem Cell and Regenerative Medicine, Institute of Science Tokyo, 1-5-45 Yushima, Bunkyo-Ku, Tokyo, 113-8510 Japan

**Keywords:** Cellular senescence, Organoid, Oxidative stress, Senescence-associated secretory phenotype, Synovial fibroblast

## Abstract

**Background:**

Osteoarthritis (OA) is a common age-related joint disease characterized by cartilage loss and synovitis. Cellular senescence contributes to chronic inflammation through senescence-associated secretory phenotypes (SASPs). Recent evidence indicates that senescent synovial fibroblasts (SFs) contribute to OA pathogenesis. However, the in vitro modeling of the microenvironment formed by senescent SFs via SASPs remains limited. Here, we developed a three-dimensional (3D) senescent synovial organoid model using senescent rat SFs.

**Methods:**

We created the senescent SFs by treating normal rat SFs in monolayer culture with hydrogen peroxide (H_2_O_2_). Senescence induction was confirmed by senescence-associated beta-galactosidase (SA-β-gal) staining, γH2AX expression, and transcriptomic analysis. Synovial organoids were then generated by embedding normal or senescent SFs in Matrigel. Transcriptomic and protein–protein interaction analyses were performed and the expression of key regulatory factors was evaluated by immunostaining. Transcriptomic profiles of the organoids were further compared with bulk RNA sequencing data from rat OA synovium and single-cell RNA sequencing data from human OA synovium. Rat chondrocytes and RAW264.7 cells were co-cultured with control or senescent organoids.

**Results:**

H_2_O_2_ treatment significantly increased SA-β-gal positivity and the expression of γH2AX and senescence-associated genes in monolayer-cultured SFs. Senescent organoids had thickened lining layers and distinct transcriptomic profiles compared to normal organoids. Enrichment analyses identified the upregulation of genes associated with extracellular matrix (ECM) organization, growth factor responses, and PI3K-Akt/MAPK signaling. Senescent organoids had a unique secretory phenotype characterized by elevated expression of ECM components, ECM-degrading enzymes, and proinflammatory factors. The expression of senescence-associated genes was greater in senescent organoids than in control organoids or H_2_O_2_-treated monolayer SFs. Protein–protein interaction analysis of senescent organoids identified central hub genes, including Il6, Tgfb1, Mmp13, and Ngf, with upregulation also confirmed at the protein level. Comparison with transcriptomic data from rat and human OA synovium revealed transcriptional similarities between senescent organoids and OA SFs in both species. Co-culture assays showed that senescent organoids decreased Sox9 expression and increased Col10a1 expression in chondrocytes, whereas they increased Cd80 expression and decreased Cd206 expression in RAW264.7 cells.

**Conclusions:**

Senescent organoids can faithfully recapitulate key features of the senescent SF microenvironment, offering an ideal platform for cellular senescence-based OA research.

**Supplementary Information:**

The online version contains supplementary material available at https://doi.org/10.1186/s41232-026-00436-2.

## Background

Osteoarthritis (OA) is a prevalent age-related joint disease manifested by progressive cartilage degradation, synovial inflammation, and osteophyte formation. OA symptoms, which can include pain, stiffness, swelling, and limited range of motion, worsen as the condition progresses [1]. Current estimates indicate that approximately one-quarter of adults aged 40 and over are affected by OA, with 86.7 million new cases occurring annually [2]. Furthermore, the prevalence rate has been on the rise in recent years [3]. Apart from surgical interventions, conservative treatments, including the administration of nonsteroidal anti-inflammatory drugs, hyaluronic acid, and corticosteroid, have been employed to alleviate symptoms. While these treatments have shown some success in managing pain, the development of drugs that effectively inhibit the structural degeneration of joints caused by OA remains an unresolved medical need.

Cellular senescence, initially characterized by Hayflick as an irreversible growth arrest following repeated cell division [4], has recently emerged as a significant therapeutic target for age-related diseases. Senescent cells are characterized by increased expression of cell cycle regulators (e.g., p16, p21, and p53), damaged DNA, and elevated activity of senescence-associated β-galactosidase (SA-β-gal) [5]. Senescent cells, despite being in a state of permanently arrested growth, remain metabolically active and secrete various proinflammatory cytokines, chemokines, and proteases. Cells with these phenotypes, known as senescence-associated secretory phenotypes (SASPs), have a deleterious influence on neighboring cells and tissues and contribute to the development and progression of age-related diseases, including OA [6, 7].

In OA, the process of cellular senescence is driven by multiple stress factors, including oxidative stress [8], mechanical overloading [9], and chronic inflammation [10], which interact in intricate ways. OA is also associated with an increased accumulation of senescent cells in the articular cartilage [11]. In particular, oxidative stress has been reported to contribute to joint dysfunction and inflammatory responses by inducing cellular aging among chondrocytes, chondrogenic progenitor cells, and osteoblasts [8, 12, 13]. Interestingly, the targeted elimination of senescent chondrocytes can attenuate OA progression [14], indicating a causal link between cellular senescence and disease severity. Furthermore, synovial fibroblasts (SFs) also undergo senescence in OA [15], and selective clearance of senescent SFs can significantly suppress OA development [16]. Notably, SF senescence is more severe and occurs earlier than chondrocyte senescence, highlighting a pivotal role of senescent SFs in OA pathogenesis. However, the niche formed by senescent SFs in OA remains poorly understood, mainly due to the lack of appropriate in vitro models.

Obtaining a deeper understanding of this niche requires the development of more advanced experimental models that can precisely reproduce the in vivo phenotype of senescent SFs. One promising approach is to use organoids, which are three-dimensional (3D) culture systems that can mimic the anatomical characteristics and physiological functions of healthy or diseased organs in vitro [17]. Synovial organoids, first established by Kiener et al. [18], can be generated by embedding SFs in Matrigel and maintaining the resulting multicellular units in floating culture. This method allows reconstruction of the characteristic two-layered structure of the synovium, consisting of the superficial lining layer and the loosely organized sublining layer beneath it. Synovial organoids can also recapitulate key physiological features, such as inflammatory responses and tissue remodeling, making them valuable models for studying disease mechanisms and developing drugs, especially for the treatment of rheumatoid arthritis [19]. However, to the best of our knowledge, no previous studies have generated synovial organoids using senescent SFs.

In the present study, we hypothesized that using SFs to construct three-dimensional “senescent synovial organoids” could faithfully recreate the microenvironment formed by senescent SFs in the OA synovium and contribute to a better understanding of OA pathogenesis and drug discovery. Our aim was to generate senescent synovial organoids from rat SFs and investigate their physiological phenotypes. We first used oxidative stress to induce cellular senescence in normal rat SFs. We then used the senescent SFs to generate senescent synovial organoids and conducted a comprehensive analysis of the disparities in the transcriptomes and regulatory networks of the senescent organoids by comparing them to organoids formed using normal SFs. We also evaluated the potential of our senescent synovial organoid model to recapitulate the microenvironment observed in the human OA synovium.

## Methods

### Isolation of rat synovial fibroblasts

This study was conducted in accordance with the institutional guidelines of Institute of Science Tokyo Animal Committee (permit no. A2022-166C) and ARRIVE guidelines. Synovial tissue was harvested from the knees of 4 Lewis rats at 10–12 weeks of age and digested using 3 mg/mL collagenase type V derived from Clostridium histolyticum (Sigma-Aldrich, Saint Louis, MO, USA) at 37 °C for 3 h with gentle agitation. The cells were filtered through a 70-μm cell strainer (Greiner Bio-One, Kremsmünster, Austria), washed with phosphate-buffered saline (PBS), seeded at a density of 2000 cells/cm^2^ in 145-cm^2^ culture dishes, and cultured in a growth medium consisting of α-modified minimum essential medium (α-MEM; Thermo Fisher Scientific, Waltham, MA, USA), 10% fetal bovine serum (FBS; Thermo Fisher Scientific), and 1% antibiotic–antimycotic solution (Thermo Fisher Scientific) at 37 °C under 5% CO_2_. Upon reaching 80–90% confluence, the cells were detached using 0.25% trypsin and 1 mM EDTA (Thermo Fisher Scientific). Cells at passage 1–2 were used as normal rat SFs.

### Senescence induction in rat synovial fibroblasts

To induce senescence using oxidative stress, normal rat SFs were plated at a density of 10,000 cells/cm^2^ and treated with 150 μM hydrogen peroxide (H_2_O_2_; Wako, Tokyo, Japan) for 2 h. The cells were then washed and cultured in a fresh growth medium without H_2_O_2_ for another 5 days. Untreated cells (controls) and cells treated with H_2_O_2_ (senescent cells) were grown as monolayers (indicated as Cont-ML and Sen-ML, respectively, in Fig. 1).

**Fig. 1:**
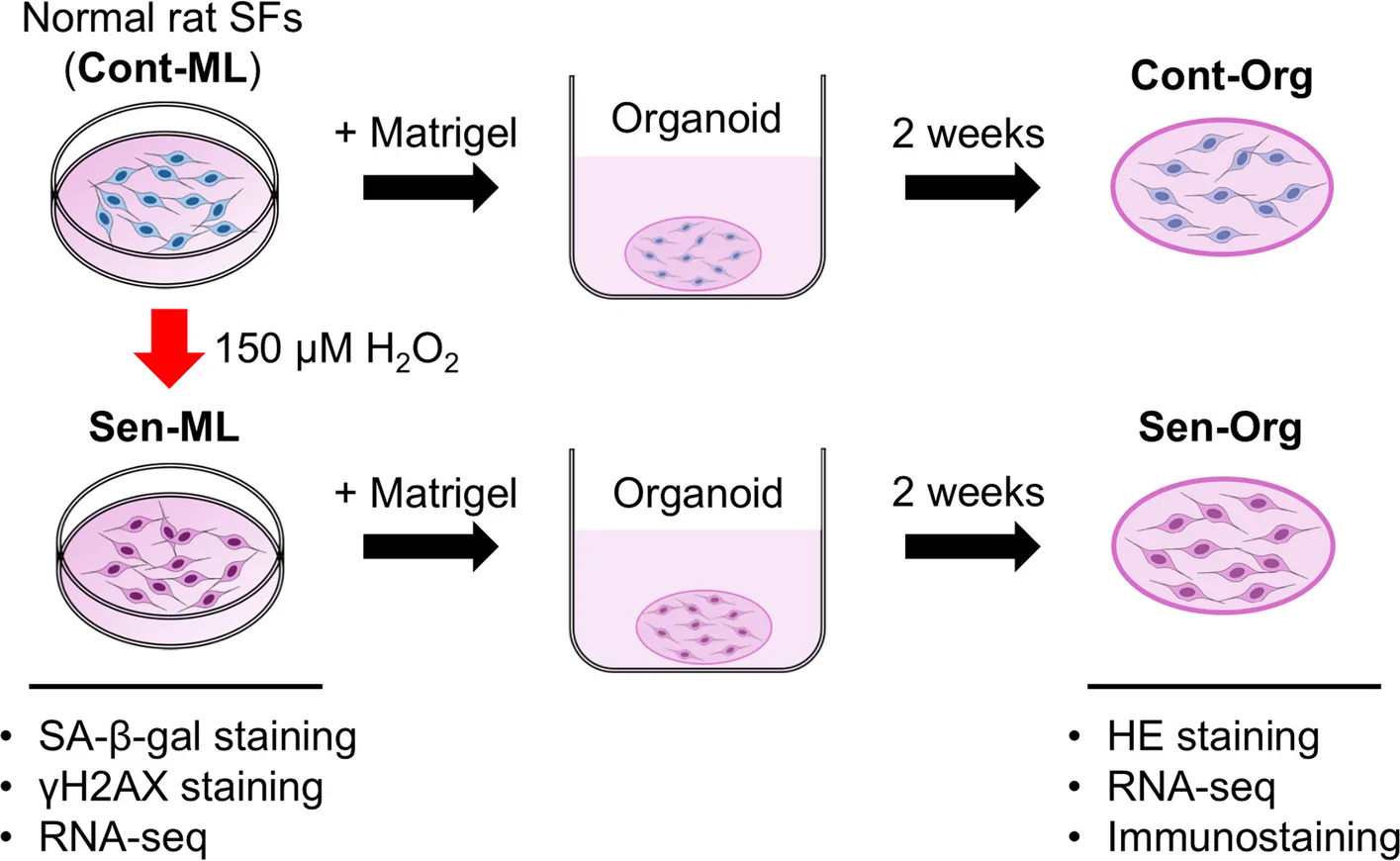
Experimental design. Normal rat synovial fibroblasts (SFs) cultured in monolayers (control-SF monolayers) were treated with 150 µM H_2_O_2_ to induce senescence (senescent-SF monolayers). These cells were subjected to senescence-associated beta-galactosidase (SA-β-gal) staining, γH2AX staining, and RNA sequencing. Organoids were generated by embedding normal or H_2_O_2_-treated SFs in Matrigel (control organoids and senescent organoids). After 2 weeks of culture, organoids were subjected to hematoxylin and eosin (HE) staining, RNA sequencing, and immunostaining. Cont-ML: untreated control SFs cultured in monolayers; Sen-ML: senescent H_2_O_2_-treated SFs cultured in monolayers; Cont-Org: untreated control SFs cultured as organoids; Sen-Org: senescent H_2_O_2_-treated SFs cultured as organoids

For senescence induction by inflammatory stress, normal rat SFs were cultured in growth medium containing 1 ng/mL recombinant rat IL-1β (PeproTech, Rocky Hill, NJ, USA) and 1 ng/mL recombinant rat TNF-α (PeproTech) for 5 days.

### Senescence-associated β-galactosidase staining

SA-β-gal staining was performed using a Senescence β-Galactosidase Staining Kit (Cell Signaling Technology, Danvers, MA, USA). Cells were fixed with fixative solution for 10 min and incubated in pH 6.0 staining solution at 37 °C for 16 h. All images were captured using a BZX700 microscope (Keyence, Osaka, Japan). The total number of cells and the number of senescence-associated β-galactosidase–positive cells were counted to calculate the senescence-associated β-galactosidase positive rate.

### Immunocytochemistry

Cells were fixed with neutral buffered 10% formalin (Wako) at room temperature and permeabilized with 0.1% Triton X-100 (Wako) in PBS for 10 min. The cells were blocked with Blocking One Histo (Nakalai Tesque, Kyoto, Japan) for 10 min and then incubated overnight at 4 °C with primary antibody against γH2AX (1:200, #9718; Cell Signaling Technology), Sox9 (1:200, AB5535; Sigma-Aldrich), Col10a1 (1:100, C7974; Sigma-Aldrich), Cd80 (1:100, bs-2211R; Bioss, Woburn, MA, USA), and Cd206 (1:500, ab64693; Abcam, Cambridge, UK). The following day, the cells were washed with Tris-buffered saline containing 0.1% Tween-20 (TBS-T) and incubated for 1 h at room temperature in the dark with an Alexa Fluor 555- or 488-conjugated secondary antibody (1:200, Abcam). Nuclei were stained with 1 μg/mL of 4′,6-diamidino-2-phenylindole (Dojindo, Kumamoto, Japan). All images were captured using a BZX700 microscope (Keyence).

### Generation of organoids

Control or senescent monolayer cells (2 × 10^5^ cells) were mixed with 15 μl of cold growth factor-reduced Matrigel (Corning, NY, USA) and seeded in 24-well plates (Corning) coated with poly 2-hydroxyethyl methacrylate (Sigma-Aldrich). After Matrigel polymerization at 37 °C for 60 min, 2 ml of growth medium was gently added to each well. Cultures were maintained for 2 weeks with medium changes every 2–3 days to enable control and senescent organoid formation (indicated as Cont-Org and Sen-Org, respectively, in Fig. 1).

### Histology

Organoids were fixed with neutral buffered 10% formalin (Wako), embedded in paraffin wax, and sliced into 5-μm sections. For hematoxylin and eosin (HE) staining, the slides were immersed in hematoxylin solution (Muto Pure Chemicals, Tokyo, Japan) for 5 min and then in eosin solution (Muto Pure Chemicals) for 3 min. For immunostaining, the slides were immersed in 10 mM Tris and 1 mM EDTA solution (pH 9.0) at 60 °C for 16 h to retrieve antigens. The slides were immersed in methanol containing 0.3% H_2_O_2_ for 30 min and then washed with TBS-T. After blocking with Blocking One Histo (NACALAI TESQUE) for 15 min, the sections were incubated overnight at 4 °C with primary antibody against Prg4 (1:200, ab28484; Abcam, Cambridge, UK), Cd90 (1:500, 66,766–1-Ig; Proteintech, Rosemont, IL, USA), Il6 (1:200, ab9324; Abcam), Tgfb1 (1:500, ab315254; Abcam), Mmp9 (1:200, 10,375–2-AP; Proteintech), Mmp13 (1:200, ab39012; Abcam), Ngf (1:200, M00341; Boster Biological Technology, Pleasanton, CA, USA), or Timp1 (1:200, 16,644–1-AP; Proteintech). After washing with TBS-T, the sections were incubated for 1 h at room temperature with HRP-conjugated secondary antibodies (1:200; Abcam). The signal was visualized using a diaminobenzidine substrate solution (Dako), and the sections were counterstained with hematoxylin. All images were captured using a BZX700 microscope (Keyence).

### RNA sequencing

Total RNA was extracted from control and senescent SF monolayers and control and senescent organoids using miRNeasy Mini Kit (Qiagen, Hilden, Germany). RNA sequencing was conducted by Azenta (South Plainfield, NJ, USA). Data were filtered to include only genes with CPM > 1 in at least 6 samples and then normalized using the edgeR package. Principal component analysis was carried out using the prcomp function. Differentially expressed genes (DEGs) were identified using the DESeq2 package with a threshold of log2 fold change (log2FC) > 0.5 and adjusted *p* value < 0.05. Heatmaps were visualized using the pheatmap package. Gene Ontology (GO) and Kyoto Encyclopedia of Genes and Genomes (KEGG) pathway analyses were conducted using the clusterProfiler package. For evaluating secretion- and senescence-associated genes, the SenMayo gene set [20] and MatrisomeDB [21] were used. In MatrisomeDB, the genes were classified into core matrisome genes (collagens, proteoglycans, and glycoproteins) and matrisome-associated genes (extracellular matrix [ECM] regulators and affiliated proteins). Gene Set Enrichment Analysis (GSEA) was performed using the GSEA software (v4.3.3; Broad Institute, Cambridge, MA, USA).

### Protein–protein interaction (PPI) network analysis

PPI analysis was performed to investigate the functional associations among the DEGs. A list of DEGs was submitted to the STRING database (v12.0) and interactions with a confidence score > 0.4 were included in the network. The resulting interaction data were imported into Cytoscape software (v3.10.3) for visualization. The node size was scaled according to the number of interactions (degree) to emphasize central or hub proteins in the network.

### Single-cell RNA sequencing

Datasets of human synovium with infrapatellar fat pads from healthy and OA donors were obtained from the Gene Expression Omnibus (GSE216651) [22]. The data were analyzed using Seurat (R, v5.2.1). After filtering out cells with poor quality (nFeature < 200, nFeature > 6000, or percent.mt > 25), the data were normalized and scaled with regressing out S.Score and G2M.Score calculated using the CellCycleScoring function. Integration was performed using harmony package. The top 30 principal components were used for uniform manifold approximation and projection (UMAP) plot, and cell clustering was performed at resolution 0.1. We annotated each cluster according to the expression of the following marker genes: VWF and PECAM1 for endothelial cells; PDGFRA and COL1A2 for SFs; ACTA2 and TAGLN for mural cells; CD14 and CD68 for macrophages; CD3D and CD3E for T cells; CPA3 and TPSAB1 for mast cells; and CD79A and CD79B for B cells. To subcluster the SFs, we extracted the SF cluster and repeated the same procedure. The SF subclusters (sublining SF, lining SF, and intermediate SF) were named according to the expression of PRG4 and THY1. The DEGs between the healthy and OA groups in each cluster were defined by min.pct > 0.1, log FC > 1, and p_val_adj < 0.05. The SenMayo score was calculated using the AddModuleScore_UCell function.

### Co-culture assays

Rat femoral and tibial articular cartilage was harvested and enzymatically digested using the same protocol described for SF isolation. After washing and filtration, the isolated cells were seeded at a density of 2000 cells/cm^2^ and expanded to obtain primary chondrocytes. The murine RAW264.7 macrophage-like cell line was purchased from ATCC (TIB-71; Manassas, VA, USA). Rat chondrocytes and RAW264.7 cells were seeded in 12-well plates at a density of 5.0 × 10^4^ cells/well in growth medium. After an overnight attachment period, transwell inserts (Corning) containing three control organoids or senescent organoids were placed in the upper chamber, and the cells were co-cultured with the organoids. Chondrocytes were co-cultured for 4 days, and RAW264.7 cells were co-cultured for 2 days. After co-culture, the cells in the lower chamber were subjected to qPCR analysis and immunocytochemical staining.

### qPCR

Total RNA was extracted using an RNeasy Mini Kit (Qiagen) and reverse-transcribed into first-strand cDNA using ReverTra Ace qPCR RT Master Mix with gDNA Remover (Toyobo, Osaka, Japan). All procedures were conducted according to the manufacturers’ instructions. Quantitative PCR was performed using Thunderbird SYBR qPCR Mix (Toyobo) on a LightCycler 480 System II (Roche Diagnostics, Basel, Switzerland). The amplification protocol consisted of 45 cycles of denaturation at 95 °C for 15 s and annealing/extension at 60 °C for 30 s. Gene expression levels were normalized to Actb or Gapdh as endogenous controls. The primer sequences used for qPCR are listed in Table S1.

### Statistical analysis

Statistical evaluations were performed using GraphPad Prism 10 (GraphPad Software, CA, USA). Comparisons between two groups were made using a two-tailed Mann–Whitney test. The significance of the DEG overlap was evaluated using Fisher’s exact test, assuming a total of 24,000 genes in both humans and rats. Values of *p* < 0.05 were considered statistically significant.

## Results

### Induction of cellular senescence in rat SF monolayers by oxidative stress

As a first step, we examined the effects of different senescence-inducing stresses on rat SFs. Oxidative stress was induced using 150 μM H_2_O_2_ and inflammatory stress was induced using IL-1β/TNF-α (IL/TNF) [23]. The H_2_O_2_-treated SFs exhibited typical senescence-associated changes, including cellular enlargement and flattening, marked growth arrest, increased SA-β-gal activity, and increased γH2AX expression (Fig. 2a). In contrast, the IL/TNF-treated SFs showed only partial growth arrest and increased SA-β-gal activity. On day 5, the number of cells in the H_2_O_2_ group remained comparable to the original number on day 0, whereas the cell numbers increased above the day 0 level in the other groups (Fig. 2b). The percentage of SA-β-gal-positive cells increased in both the H_2_O_2_ and IL/TNF groups (Fig. 2c). These findings suggest that H_2_O_2_ induced a more robust senescent phenotype, whereas IL/TNF stimulation induced a partial or less mature senescence-like state. Therefore, H_2_O_2_ treatment was used to induce senescence in SFs in all subsequent experiments.

**Fig. 2:**
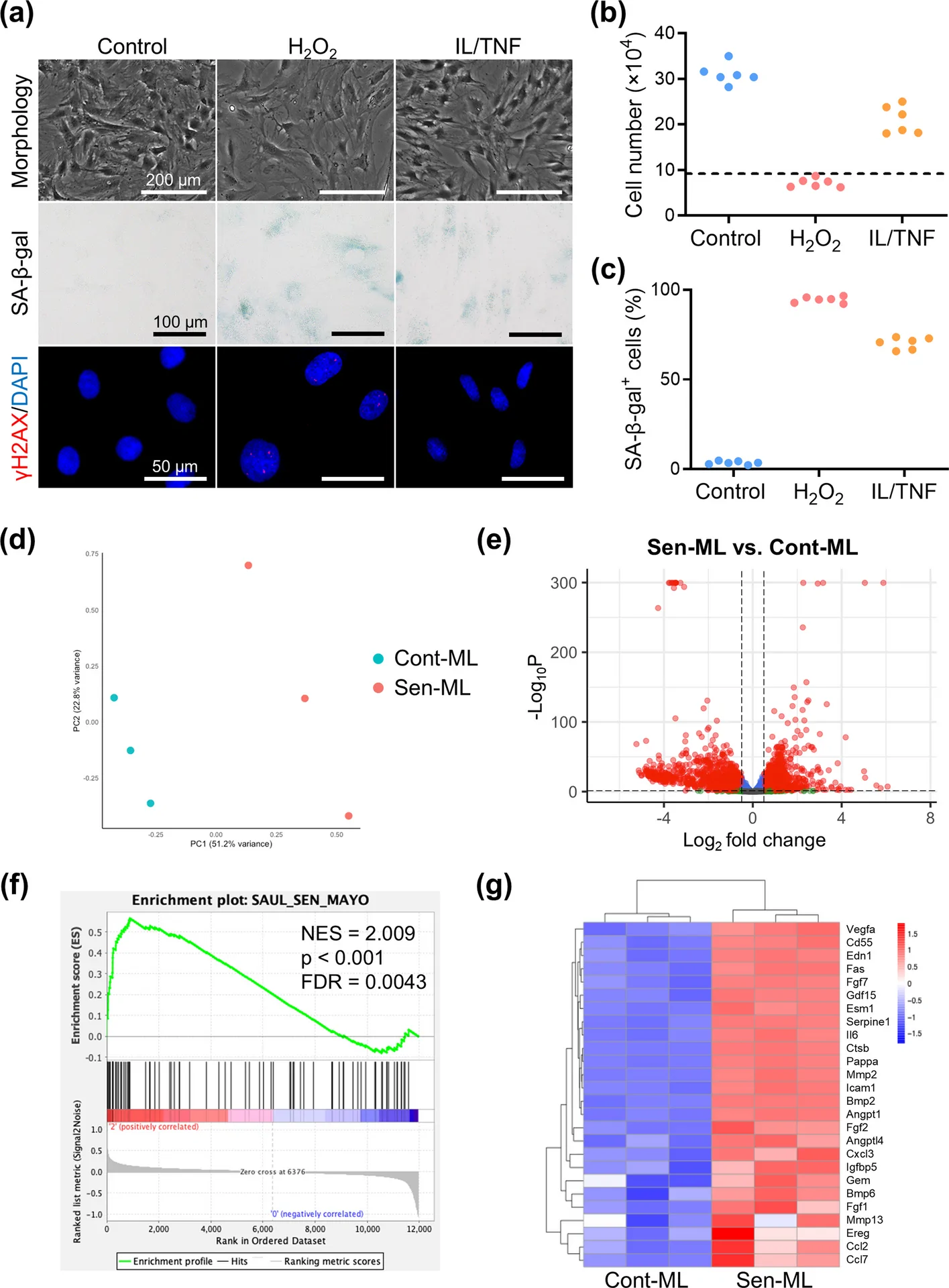
Senescent phenotypes of H_2_O_2_-treated rat synovial fibroblasts (SFs) cultured as a monolayer. **a** Representative images of cell morphology, senescence-associated beta-galactosidase (SA-β-gal) staining, and γH2AX immunostaining in control, H_2_O_2_-treated, and IL-1β and TNF-α (IL/TNF)-treated SFs. **b** The number of cells on day 5. The dotted line indicates the number of cells on day 0 (*n* = 6). **c** Percentages of SA-β-gal-positive cells (*n* = 6). **d** Principal component analysis of control-SF monolayer and senescent-SF monolayer transcriptomes. **e** Volcano plots of differentially expressed genes (DEGs) between senescent-SF monolayers and control-SF monolayers. **f** Gene Set Enrichment Analysis of SenMayo in senescent-SF monolayers vs. control-SF monolayers. NES, normalized enrichment score; FDR, false discovery rate. **g** Heatmap of SenMayo genes upregulated in senescent-SF monolayers vs. control-SF monolayers. Cont-ML: untreated control SFs cultured in monolayers; Sen-ML: senescent H_2_O_2_-treated SFs cultured in monolayers

We used RNA sequencing to assess the transcriptomic changes in senescent H_2_O_2_-treated SFs cultured in monolayers. Principal component analysis revealed a clear segregation of control-SF monolayers and senescent-SF monolayers (Fig. 2d). Compared with control-SF monolayers, 1395 genes were upregulated and 1254 genes were downregulated in senescent-SF monolayers (Fig. 2e). Heatmap analysis of the DEGs visualized distinct gene expression profiles between the groups, supported by hierarchical clustering (Fig. S1a). GO enrichment analysis showed that DEGs upregulated in senescent-SF monolayers were enriched in processes such as “chemotaxis” and “response to interleukin-1” (Fig. S1b). KEGG pathway analysis indicated that the upregulated DEGs were involved in rheumatoid arthritis (Fig. S1c). GSEA using SenMayo, a senescence-associated gene set, showed a significant upregulation of senescence-associated genes in senescent-SF monolayers (Fig. 2f). Among the SenMayo gene set, 26 genes, including Il6, Mmp2, and Mmp13, overlapped with DEGs upregulated in senescent-SF monolayers (Fig. 2g).

### Generation of synovial organoids and their transcriptome characterization

Normal and senescent synovial organoids were generated by embedding untreated control or H_2_O_2_-treated rat SFs in Matrigel and culturing them for 2 weeks. HE staining revealed that the surface of the normal organoids was composed of 1–2 layers of SFs, whereas the SFs formed a thicker lining layer on the surface of the senescent organoids (Fig. 3a). In both the normal and senescent organoids, Prg4 immunostaining was predominantly detected on the surface, whereas Cd90 immunostaining was weak at the surface and was mainly observed in cells located in the internal zone (Fig. S2).

**Fig. 3:**
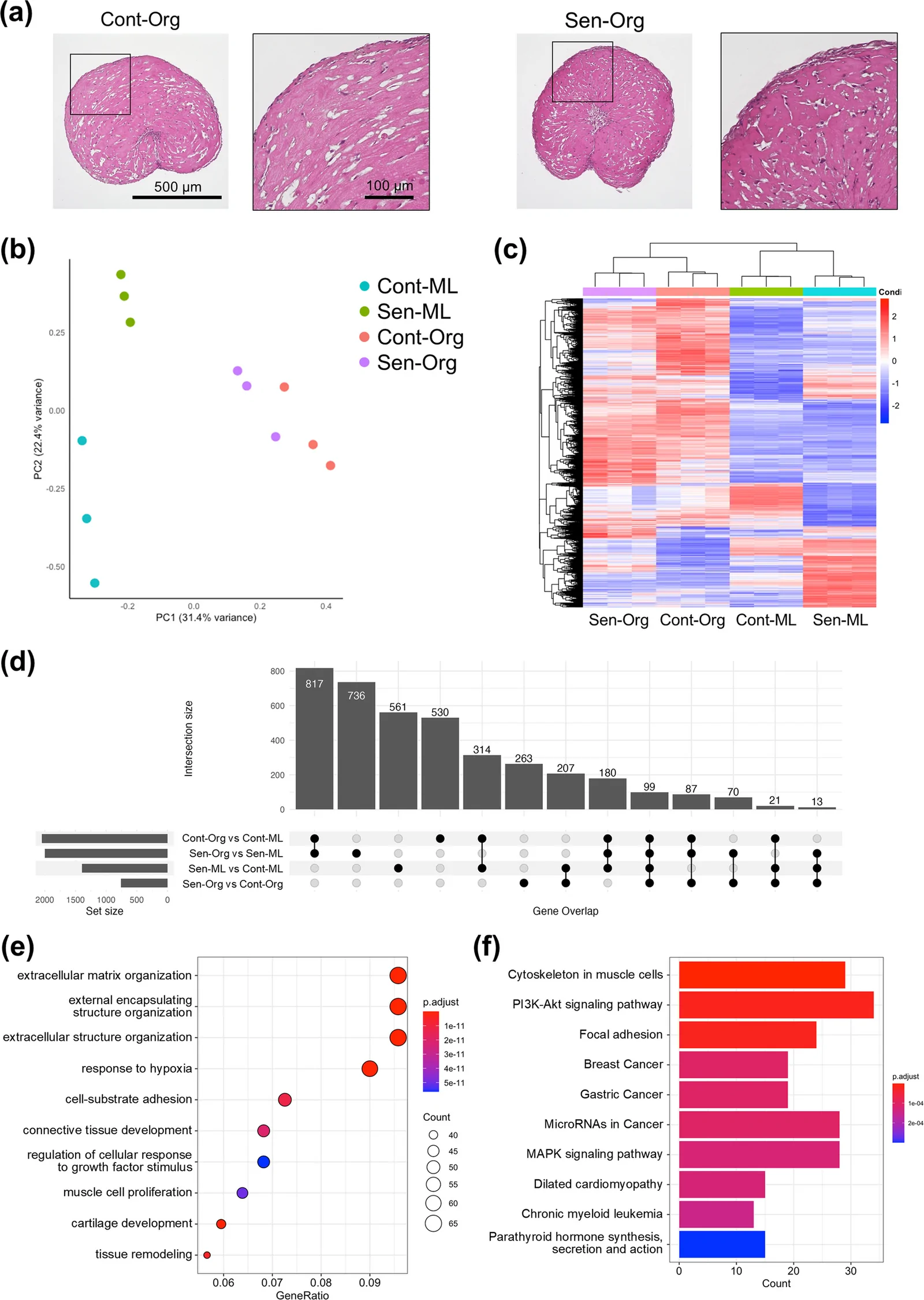
Transcriptome characterization of synovial organoids. **a** Representative images of the hematoxylin and eosin staining of organoids. **b** Principal component analysis of control-SF monolayers, senescent-SF monolayers, control organoids, and senescent organoids. **c** Gene expression heatmap of differentially expressed genes (DEGs). **d** Upset plot of DEGs. **e** Gene ontology analysis of 760 DEGs upregulated in the senescent organoids vs. control organoids. The top 10 terms are shown as a bubble plot. **f** Kyoto Encyclopedia of Genes and Genomes pathway analysis of 760 DEGs upregulated in the senescent organoids vs. control organoids. The top 10 terms are shown as a bar chart. Cont-ML: untreated control SFs cultured in monolayers; Sen-ML: senescent H_2_O_2_-treated SFs cultured in monolayers; Cont-Org: untreated control SFs cultured as organoids; Sen-Org: senescent H_2_O_2_-treated SFs cultured as organoids

RNA sequencing of the control-SF and senescent-SF monolayers and organoids and subsequent PCA plot and hierarchical clustering demonstrated closer clustering between the organoids than between the monolayer cultures (Fig. 3b, c). We identified 2048 DEGs for the control organoids vs. control-SF monolayers, 2002 for the senescent organoids vs. senescent-SF monolayers, 1395 for the senescent-SF monolayers vs. control-SF monolayers, and 760 for the senescent organoids vs. control organoids (Fig. 3d and Fig. S3). Among them, 817 DEGs were common to control organoids vs. control-SF monolayers and senescent organoids vs. senescent-SF monolayers. DEGs upregulated in the senescent organoids vs. control organoids were related to ECM organization, response to growth factors, and tissue development/remodeling in GO analysis (Fig. 3e), and PI3K-Akt signaling, MAPK signaling, and various cancer pathways in KEGG analysis (Fig. 3f).

### Secretion- and senescence-associated gene expression in synovial organoids

The results of GO analysis led us to compare the ECM/soluble factor secreting phenotype of synovial organoids using MatrisomeDB. Among the DEGs upregulated in senescent organoids vs. control organoids, 38 genes overlapped with the core matrisome, which contains essential structural elements of the ECM (Fig. 4a). These included various collagens and some glycoproteins. In addition, 80 genes were overlapped with matrisome-associated genes consisting of ECM-affiliated proteins, ECM regulators, and secreted factors (Fig. 4b). Typical examples of these genes included Il6/7/16/34, Tgfb1/2/3, Mmp2/9/13/16/25, Timp1/3, Vegfa/c, and Ngf. Some of these genes overlapped with an SASP gene set previously defined by Binet et al. [24]. We also used SenMayo to interrogate the DEGs upregulated in senescent organoids vs. control organoids to assess the senescent phenotype. Seventeen genes (Il6/7, Mmp2/9/13/14, Vegfa/c, Angpt1/4, Bmp6, Ccl7, Fgf1, Gdf15, Icam1, Pappa, and Pgf) were identified as overlapping genes (Fig. 4c). GSEA revealed a significant enrichment of SenMayo genes in senescent organoids compared to control organoids or senescent-SF monolayers (Fig. 4d). Recent studies have reported increased expression of Il1a, Cdkn1a, Cdkn2a, Angptl4, and Rcan1 in senescent SFs in human OA [25, 26]. Therefore, we also examined the expression of these genes in our model. Among these genes, Cdkn1a was upregulated and Angptl4 was markedly upregulated in the senescent synovial organoids, whereas Il1a, Cdkn2a, and Rcan1 did not show increased expression (Fig. S4).

**Fig. 4:**
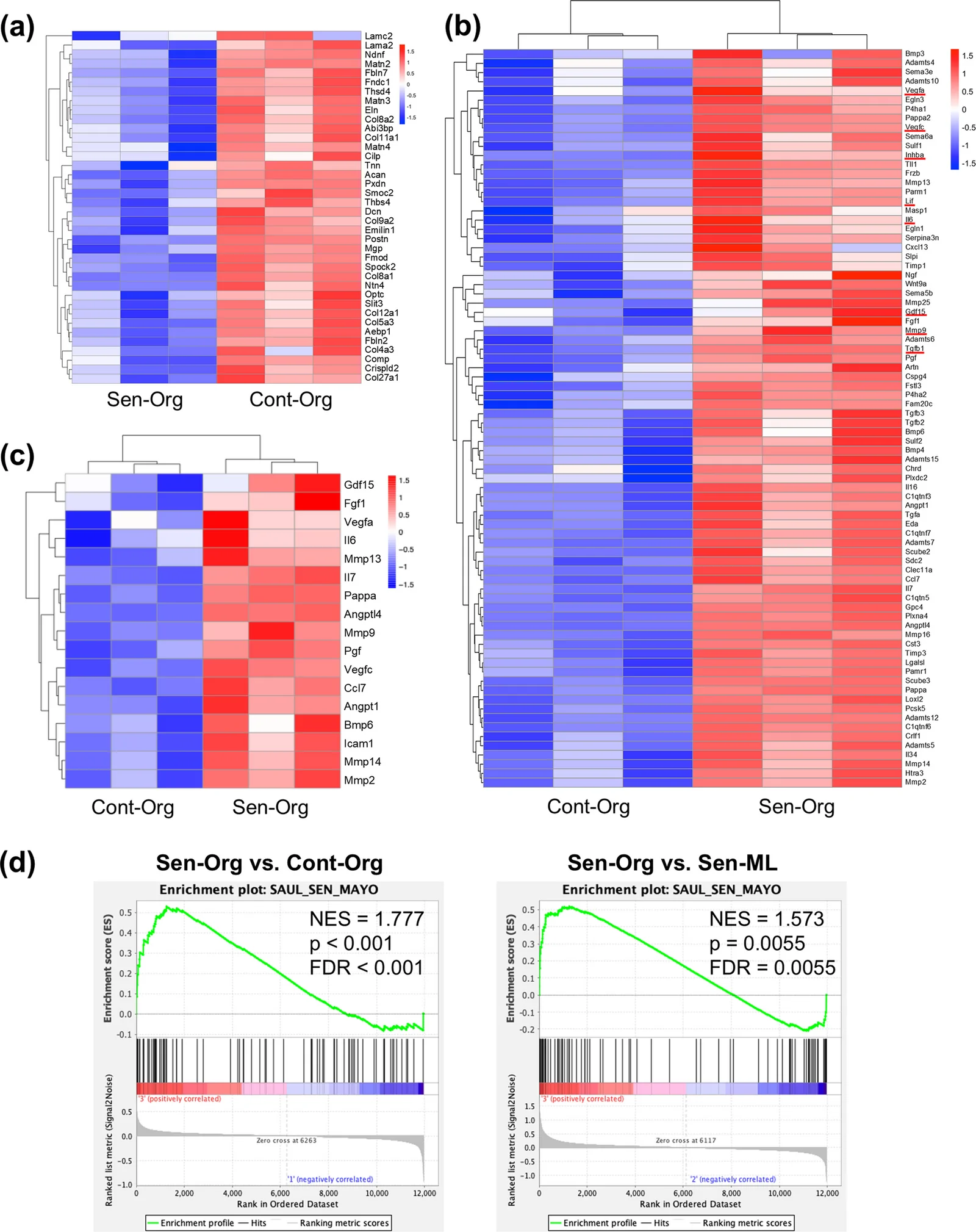
Secretion and senescence phenotypes of synovial organoids. **a** Heatmap of core matrisome genes upregulated in senescent organoids vs. control organoids. **b** Heatmap of matrisome-associated genes upregulated in senescent organoids vs. control organoids. Red underlines indicate genes that overlap with the SASP_Curated_UP gene set. **c** Heatmap of SenMayo genes upregulated in senescent organoids vs. control organoids. **d** Gene Set Enrichment Analysis of SenMayo in senescent organoids vs. control organoids and senescent organoids vs. senescent monolayers. Sen-ML: senescent H_2_O_2_-treated SFs cultured in monolayers; Cont-Org: untreated control SFs cultured as organoids; Sen-Org: senescent H_2_O_2_-treated SFs cultured as organoids; NES, normalized enrichment score; FDR, false discovery rate

### Key genes in synovial organoids generated from H_2_O_2_-treated SFs

To explore functional interactions among the DEGs upregulated in senescent organoids vs. control organoids, we performed PPI analysis using the STRING database. We identified 525 interacting genes and visualized the network using the top 40 genes by number of interactions (Fig. 5a). Il6 had the greatest number of interactions with other genes. Other genes with high interaction count included Tgfb1, Mmp9/13, Ngf, and Timp1 (Table S2). Immunostaining revealed higher expression of Il6, Tgfb1, Mmp13, and Ngf in senescent organoids than in control organoids. Tgfb1 expression was localized to the lining layer of senescent organoids. Mmp9 and Timp1 showed no apparent increase in staining intensity (Fig. 5b).

**Fig. 5:**
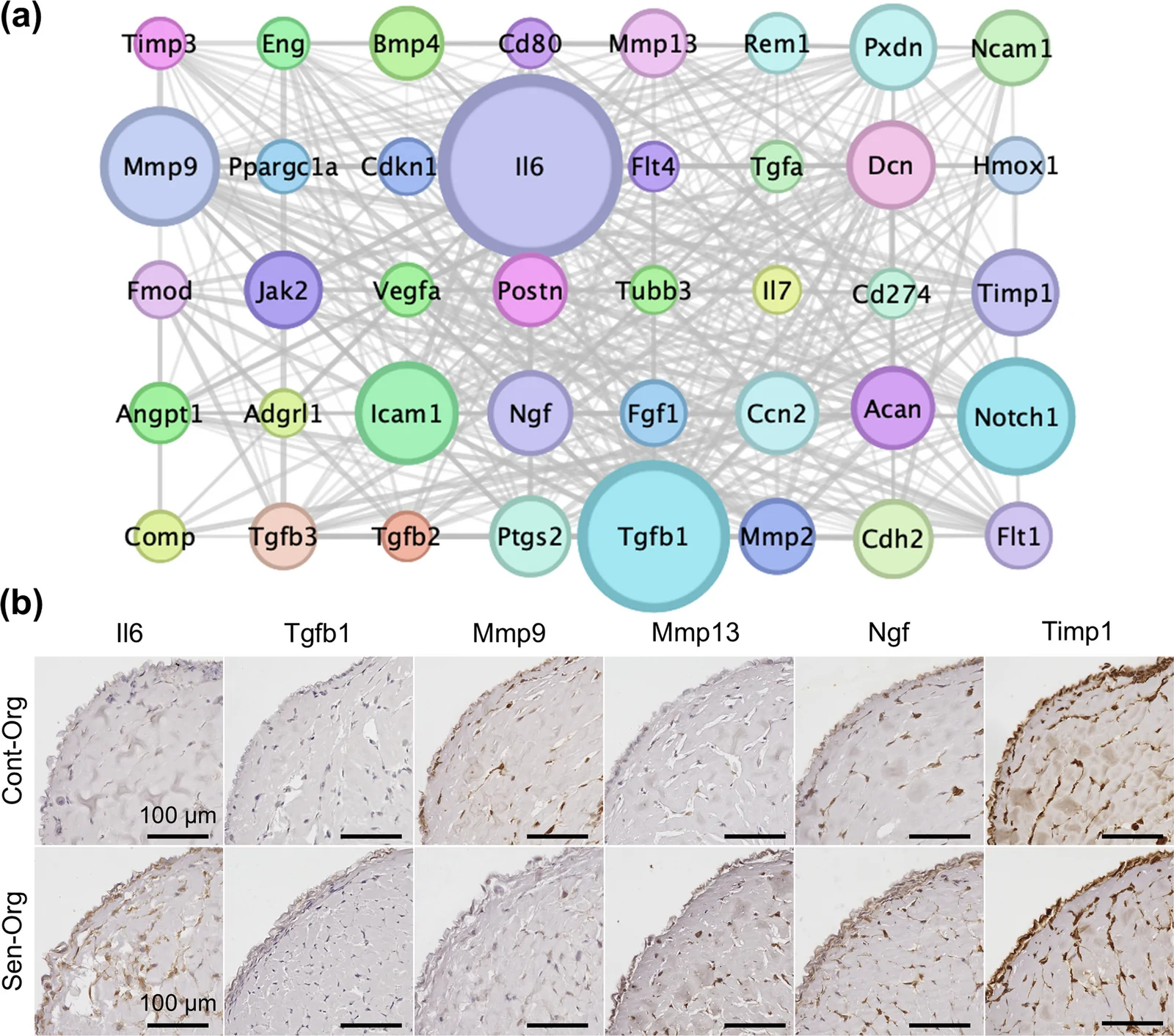
Key regulatory genes in synovial organoids generated from H_2_O_2_-treated rat synovial fibroblasts. **a** Protein–protein interaction analysis of genes upregulated in senescent organoids vs. control organoids. The top 40 genes with the highest number of interactions are visualized by Cytoscape. The size of each node is proportional to the number of interactions. **b** Representative images of immunostaining for Il6, Tgfb1, Mmp9, Mmp13, Ngf, and Timp1 in control organoids and senescent organoids. Cont-Org: untreated control SFs cultured as organoids; Sen-Org: senescent H_2_O_2_-treated SFs cultured as organoids

### Comparison with RNA sequencing data from rat OA synovium

We evaluated the transcriptomic similarity between the senescent organoids generated in this study and synovium from a rat OA model by comparing our data with DEG data obtained for rat synovium collected at 2, 4, and 6 weeks after medial meniscus transection (MMT) surgery [27]. Compared with the sham group, the MMT rats showed upregulation of 28, 63, and 13 genes in the synovium at 2, 4, and 6 weeks, respectively. Among these upregulated genes, 10, 18, and 4 genes overlapped with the 760 genes identified as upregulated in senescent organoids vs. control organoids (Fig. 6). These overlaps were statistically significant at all time points, with the strongest enrichment observed at 4 weeks after MMT surgery.

**Fig. 6:**
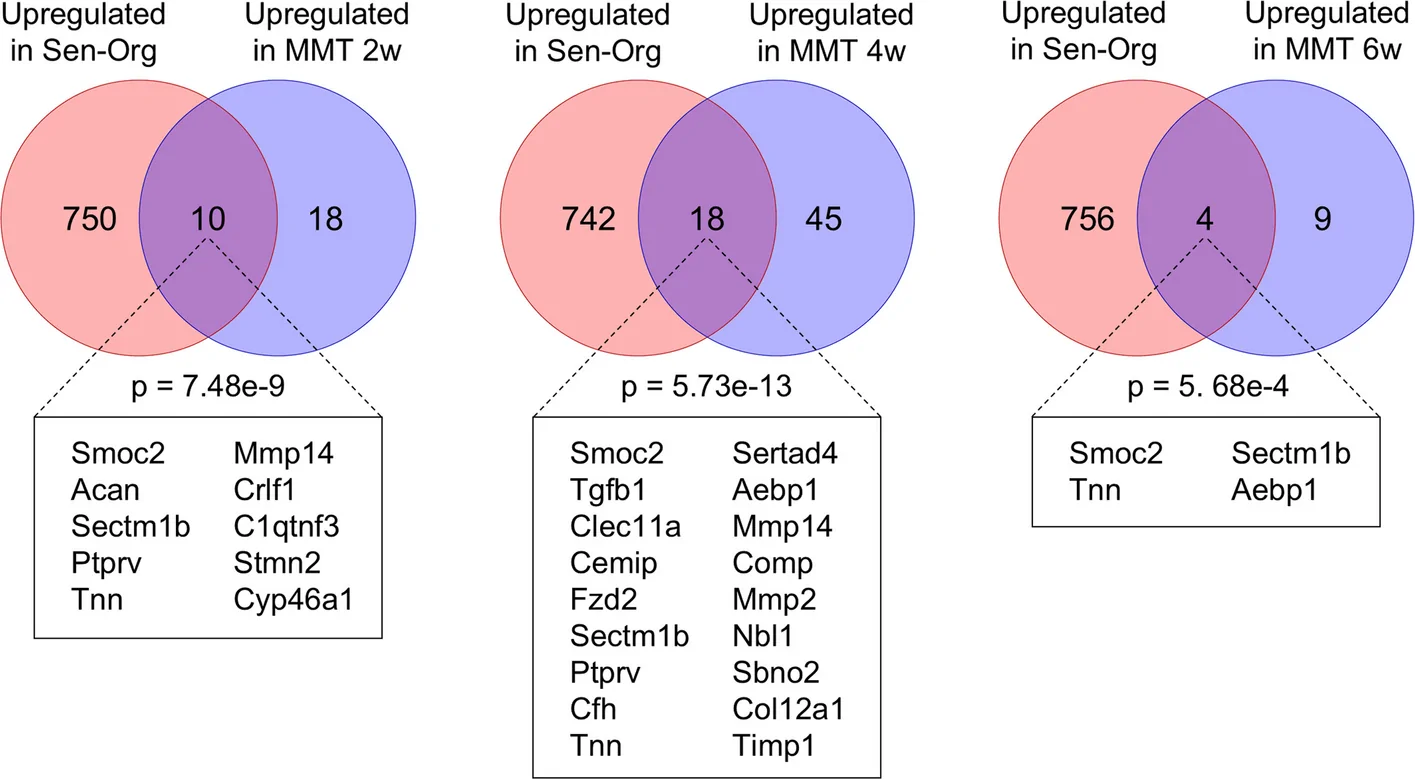
Transcriptomic similarity between rat osteoarthritic (OA) synovium and rat synovial organoids. Overlap of genes upregulated at 2, 4, and 6 weeks after medial meniscus transection (MMT) surgery vs. sham controls and genes upregulated in senescent organoids vs. control organoids. The overlapping genes are shown in the boxed area. Sen-Org: senescent H_2_O_2_-treated SFs cultured as organoids

### Comparison with single-cell RNA sequencing data of human OA synovium

We tested the similarities in transcriptomes between senescent organoids generated in the present study and the human OA synovium using deposited single-cell RNA sequencing datasets from healthy and OA synovium [22]. We classified 67,278 cells into seven cell types (endothelial cells, SFs, mural cells, macrophages, T cells, mast cells, and B cells) according to marker gene expressions (Fig. S5a, b). The SFs (35,511 cells) were extracted and re-clustered into the following three subsets: sublining SF, lining SF, and intermediate SF (Fig. 7a). We evaluated the enrichment of senescent gene signatures between healthy and OA SFs by calculating the SenMayo score for each cell. All SF subsets showed significantly higher SenMayo scores in the OA group than in the healthy group (Fig. 7b). We then analyzed the DEGs upregulated in the OA vs. healthy groups in each SF subset (Table S3) and interrogated them with the SenMayo genes. Although some differences were apparent between the SF subsets, many senescence-related genes, including IL6, were highly expressed in the OA synovium (Fig. 7c). Finally, to verify whether senescent organoids have a similar signature to that of OA SFs, we compared the DEGs upregulated in OA vs. healthy in each SF subset to the DEGs upregulated in senescent organoids vs. control organoids. The DEGs in senescent organoids were significantly overlapped with the DEGs upregulated in the OA sublining (70 genes), lining (62 genes), and intermediate SF (62 genes) (Fig. 7d). Construction of the PPI network using the DEGs from each subset revealed hub genes, such as IL6, TGFB2/3, and TIMP1, that were common to the network in senescent-SF monolayers (Fig. S6a, b, c).

**Fig. 7:**
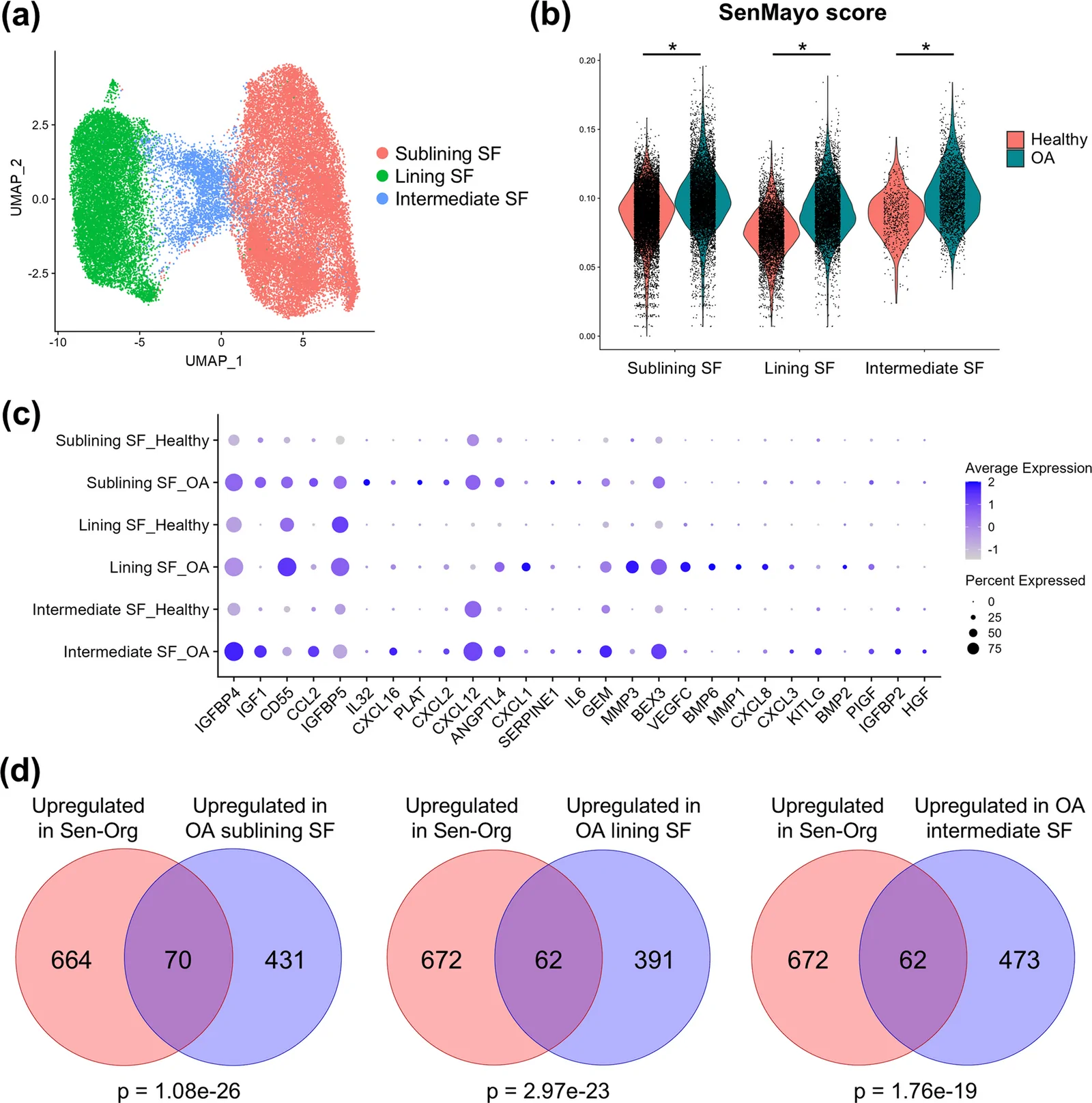
Transcriptomic similarity between human osteoarthritic (OA) synovial fibroblasts (SFs) and rat synovial organoids. **a** Uniform manifold approximation and projection plot showing human SF subsets. **b** Violin plot showing the SenMayo score of each SF subset of human healthy and OA donors. **c** Dotplot of SenMayo genes upregulated in OA SF. **d** Overlap of genes upregulated in OA vs. healthy in each SF subset and genes upregulated in senescent organoids vs. control organoids. Sen-Org: senescent H_2_O_2_-treated SFs cultured as organoids

### Functional evaluation of synovial organoids using co-culture assays

We evaluated the functional effects of senescent synovial organoids on cartilage degeneration and inflammatory regulation by performing transwell co-culture assays using primary rat chondrocytes and RAW264.7 macrophage-like cells (Fig. 8a). Primary chondrocytes co-cultured with senescent synovial organoids showed decreased gene expression of Sox9, a master regulator for chondrogenesis, and increased expression of a hypertrophic marker Col10a1 when compared with primary chondrocytes co-cultured with control organoids (Fig. 8b). These changes were supported by immunocytochemical staining, which showed a reduced nuclear Sox9 signal and the appearance of Col10a1-positive cells in chondrocytes co-cultured with senescent organoids (Fig. 8c). RAW264.7 cells co-cultured with senescent organoids showed an increasing trend in the gene expression of M1 markers Cd80 and Tnf, whereas the M2 markers Cd206 and Arg1 were decreased (Fig. 8d) when compared with RAW264.7 cells cultured with control organoids. The Cd80/Cd206 ratio, an indicator of the M1/M2 macrophage balance, was markedly increased after co-culture with senescent organoids. Consistent with these gene expression changes, immunocytochemical staining showed enhanced CD80 expression and reduced CD206 expression in cells exposed to senescent organoids (Fig. 8e).

**Fig. 8:**
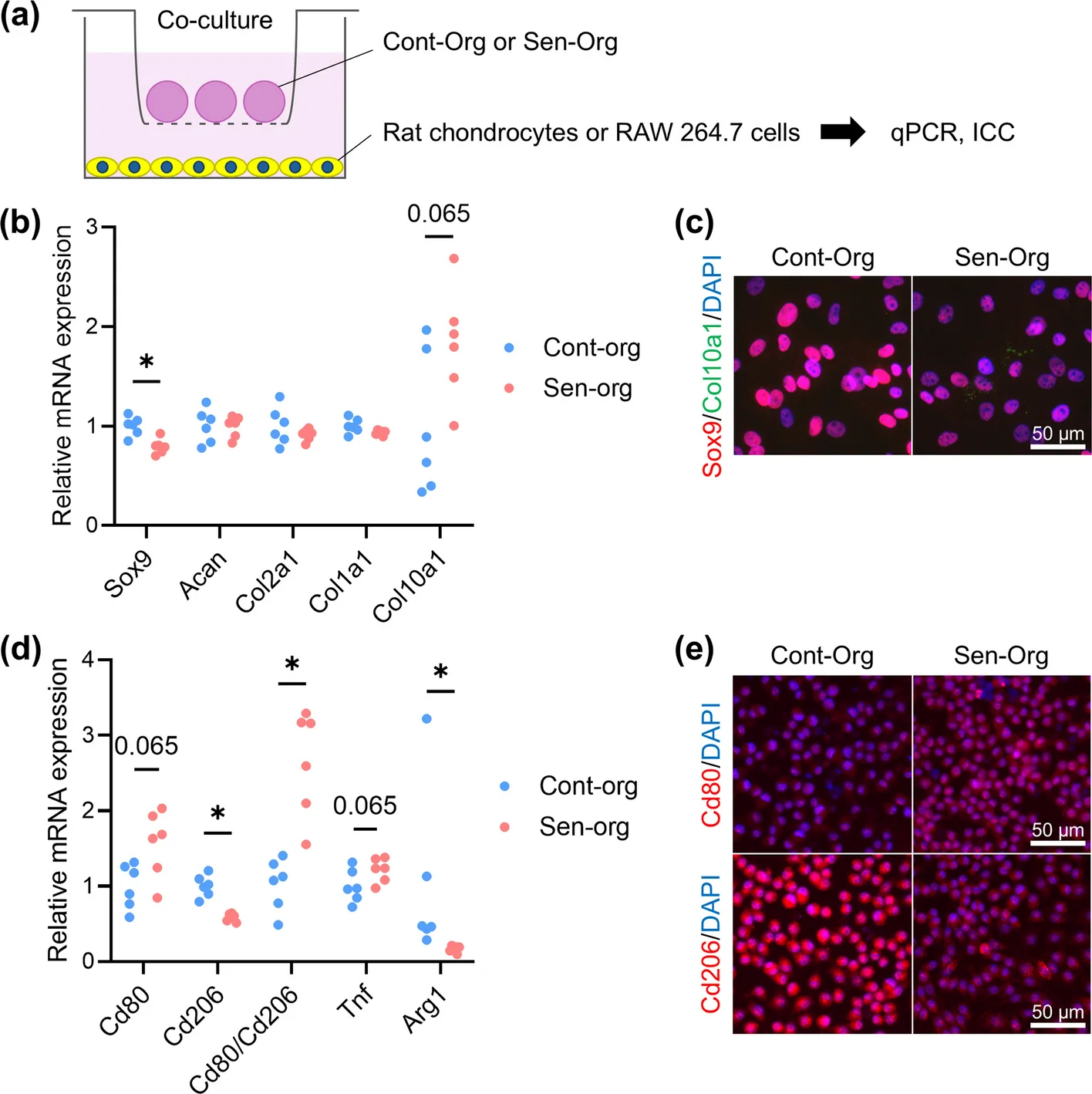
Functional evaluation of synovial organoid effects in co-culture assays. **a** Schematic illustration of the experiments. Rat primary chondrocytes or RAW264.7 cells were seeded in the lower chamber, and transwell inserts containing three control organoids or senescent organoids were placed. Chondrocytes were co-cultured for 4 days, and RAW264.7 cells were co-cultured for 2 days. **b** qPCR analysis of chondrocytes after co-culture (*n* = 6). *p < 0.05. **c** Representative immunocytochemical images of Sox9 and Col10a1 expression in chondrocytes after co-culture. **d** qPCR analysis of RAW264.7 cells after co-culture (*n* = 6). *p < 0.05. **e** Representative immunocytochemical images of Cd80 and Cd206 expression in RAW264.7 cells after co-culture. Cont-Org: untreated control SFs cultured as organoids; Sen-Org: senescent H_2_O_2_-treated SFs cultured as organoids

## Discussion

Monolayer-cultured rat SFs treated with H_2_O_2_ exhibited classical features of cellular senescence, including a large and flat morphology and increased SA-β-gal activity. GO analysis revealed enrichment of genes associated with chemotaxis and responses to interleukin-1 in the senescent-SF monolayers, together with high expression of various chemokines and inflammatory cytokines, reflecting the fact that the SASP factors released from senescent cells are known to mobilize and activate immune cells [28]. Additionally, the senescent-SF monolayers showed elevated expression of genes associated with rheumatoid arthritis, corroborating a previously identified association between cellular senescence and the pathogenesis of rheumatoid arthritis [29].

The GSEA findings presented here demonstrated a significant upregulation of the SenMayo gene set in senescent-SF monolayers, supporting the successful induction of cellular senescence by treating rat SFs with H_2_O_2_. Montero-Melendez et al. have previously triggered senescence in human SFs using BMS-470539, a melanocortin-1 receptor agonist [30]. However, their BMS-470539 treatment did not induce proinflammatory SASPs, such as IL6 and CCL2. In the present study, H_2_O_2_ treatment resulted in substantial upregulation of these SASP factors, suggesting that oxidative stress can induce a more disease-relevant and proinflammatory form of senescence.

We tested this possibility further by comparing our generated synovial organoids containing normal and senescent SFs. Consistent with a previous study of human synovial organoids [18], our rat synovial organoids developed a distinct lining–layer structure. Immunostaining revealed that our organoids contained cells positive for Prg4, a lining SF marker, and Cd90, a sublining SF marker [31], with predominant localization at the surface and in the internal zone, respectively. These findings further support the emergence of SF states with distinct phenotypes and suggest partial recapitulation of synovial tissue architecture. Interestingly, our rat senescent organoids formed a thicker lining layer that phenotypically resembled the synovial hyperplasia observed in OA [32]. Transcriptomic profiling revealed that the rat SFs underwent dynamic reprogramming during organoid formation, as previously reported for other organoid systems [33, 34]. This is likely attributable to exposure of the SFs to a more physiologically relevant environment due to an altered matrix and changes in signaling when grown in 3D culture. The differences in DEGs upregulated in senescent organoids vs. control organoids indicated an enrichment in genes related to ECM organization, including various types of collagens.

In OA, the SFs acquire a myofibroblast-like phenotype and contribute to synovial fibrosis [35] through mechanisms that are not yet fully understood. Our findings suggest that this senescent organoid model may provide insights into the molecular basis of SF behavior in fibrosis, as KEGG analysis identified further enrichment of genes involved in the PI3K-Akt and MAPK signaling pathways. These pathways are associated with OA pathogenesis [36, 37], but they also play a central role in the regulation of cellular senescence and SASP [38]. Pathways associated with various cancers were also enriched in senescent organoids, supporting the function of cellular senescence as a promoter of tumorigenesis [39].

The interrogation of DEGs using the MatrisomeDB revealed the upregulation of core matrisome components (e.g., collagens, Dcn, and Acan) and matrisome-associated genes (e.g., Adamts4/5, Mmp9/13, Il6, Ngf, and Tgfb1) in the senescent-SF monolayers. The expression of the core matrisome is known to be specific to the cell type and differentiation state, and alterations in these genes have been implicated in the pathology of degenerative diseases [40]. Since SF senescence is considered to precede cartilage degeneration in OA [16], the core matrisome genes identified in this study might serve as potential therapeutic targets or biomarkers, particularly for early-stage OA. With regard to matrisome-associated genes, the expression levels of OA-related factors, which include Mmp9/13 [41], Tgfb1 [42] and Il6 [43], were significantly elevated in senescent-SF monolayers. GSEA using SenMayo gene set demonstrated that the senescent organoids shifted toward a deeper senescence state when compared to control organoids and senescent-SF monolayers, indicating the utility of organoids in recapitulating typical OA and senescence phenotypes. Furthermore, Angptl4 was robustly upregulated in our senescent synovial organoids. Since Angptl4 has recently been implicated in senescent SF-mediated OA pathology [26], this finding suggests that our model captures at least one disease-relevant feature of OA-associated SFs.

Among the hub genes identified by PPI analysis, Il6, Tgfb1, Mmp13, and Ngf were highly expressed at the protein level in the senescent organoids. Il6, which formed the strongest interaction with other genes, is known to function as a key hub gene in human knee OA [44]. Other studies have also explored the relationship between Il6 and OA [43, 45]. Our senescent synovial organoids may therefore be useful in studies of OA pathophysiology and drug discovery, especially those focused on Il6. Similarly, Tgfb1, which is traditionally known as an essential factor for chondrogenesis, plays complex roles in OA development and progression [46]. In particular, excessive Tgfb1 activity induces synovial hyperplasia and fibrosis by promoting SF proliferation and ECM deposition. Likewise, Mmp13 is a key enzyme in type II collagen and proteoglycan degradation and its serum level correlates with OA severity [47], while Ngf, which contributes to inflammation and pain via neuropeptide signaling and angiogenesis [48], is associated with OA progression [49].

We validated the in vivo and translational relevance of our senescent organoids by comparing their transcriptomic profiles with rat and human OA synovium. Significant overlap was detected between the DEGs that were upregulated in senescent organoids and those upregulated in rat MMT synovium at all the examined time points. The strongest enrichment was observed at 4 weeks post-MMT. This timing is significant because the acute-phase response in the rat MMT model reportedly occurs at 1–3 weeks and is followed by a chronic-phase response between 6 and 12 weeks [50]. Thus, the marked overlap at 4 weeks suggests that our senescent organoids reflect transcriptomic features associated with the progressive phase of OA. Furthermore, when compared with single-cell human synovial RNA sequencing data, each SF subset had significantly higher SenMayo scores in the OA than in the healthy group, supporting the progression of cellular senescence in the OA synovium [14]. DEGs upregulated in senescent organoids vs. control organoids significantly overlapped with those upregulated in the OA vs. healthy groups in each SF subset, and they shared hub genes, such as IL6. These findings indicate that our senescent organoids effectively recapitulate transcriptomic and pathological features of both rat and human OA synovium and could serve as a valid tool for studying OA pathogenesis.

We also assessed the functional relevance of senescent synovial organoids. Notably, co-culture with senescent organoids reduced Sox9 expression and increased Col10a1 expression in chondrocytes, indicating that soluble factors released from senescent organoids can induce a shift in the articular cartilage toward a more hypertrophic or degenerative phenotype. In RAW264.7 cells, co-culture with senescent organoids enhanced Cd80 expression while suppressing Cd206 expression, resulting in a marked increase in the Cd80/Cd206 ratio. These reciprocal changes demonstrate that senescent organoids exacerbated the M1/M2 polarization balance and induced a more proinflammatory phenotype. These functional observations are consistent with the previous reports [25, 26] and support the idea that senescent synovial organoids do not merely reproduce senescence-associated gene expression. Rather, senescent organoids can also induce cartilage and immune cells to undergo changes that are relevant to OA pathology.

Overall, our results demonstrate the successful establishment of a novel senescent synovial organoid model that recreates the senescent SF niche in OA. Moreover, as far as we know, this is the first study to create senescent organoids using senescent cells. Our organoid model contributes to the understanding OA pathogenesis while also holding significant promise as a drug discovery platform for evaluating and screening new OA therapeutics. The recent report of direct generation of synovial organoids from SFs of OA patients [25] is a valuable advance, as patient-derived organoids are expected to reflect the complex pathological environment of human OA. However, these directly derived organoids may also be influenced by multiple disease-related and patient-specific factors, including chronic inflammation, mechanical stress, aging, donor variability, and medication history. Our model avoided this complication, as senescence was induced in normal SFs before they underwent 3D culture, thereby allowing evaluation of senescence-associated changes in the 3D synovial microenvironment under more controlled experimental conditions. We anticipate that this controllable system may be particularly useful for dissecting the contributions of SF senescence to OA-related synovial pathology. Notably, the observed similarity in gene expression patterns to those occurring in the human OA synovium, together with the reproducible expression of proinflammatory SASP factors, suggests that this model could be particularly useful for developing OA treatments that target cellular senescence. Future studies to incorporate other synovial components, such as vascular endothelial cells, macrophages, and neurons, into organoids should enable more precise reconstruction of the complicated OA synovial microenvironment [31], thereby enhancing the accuracy and versatility of our model. Furthermore, establishing this model using human senescent SFs may provide insights with greater clinical relevance.

The present study has two main limitations. First, we used rat SFs because obtaining normal/nonsenescent cells was easy. However, rat SFs may differ from human SFs in terms of gene signatures and biological responses. Future studies should aim to develop synovial organoids using human SFs. Second, we induced cellular senescence by direct treatment with H_2_O_2_, but the chronic aging process of the knee joints is driven by a range of stressors. Thus, the responses occurring during aging might not be fully replicated by acute oxidative stress alone.

## Conclusions

This study presents a novel senescent synovial organoid consisting of senescent SFs from rats. The H_2_O_2_ treatment used here induced cellular senescence in monolayer-cultured rat SFs, as evidenced by the upregulation of SA-β-gal activity and senescence-associated gene expression. When compared to synovial organoids generated from unstressed normal SFs, the synovial organoids generated from these senescent SFs formed a thicker lining layer and exhibited increased expression of genes associated with ECM organization, tumorigenesis, cellular senescence, and SASPs. OA-related factors, such as Il6, Tgfb1, Mmp13, and Ngf, were identified as key regulators of the microenvironment formed by the senescent SFs in organoids. The organoids generated from senescent SFs had transcriptomic features similar to those observed in both rat and human OA synovium. Notably, the co-culture with senescent synovial organoids promoted degenerative and hypertrophic changes in chondrocytes and proinflammatory polarization in macrophages. Our findings indicate that senescent synovial organoids can faithfully recapitulate the OA synovial microenvironment, thereby opening up new possibilities for OA drug discovery research targeting cellular senescence.

## Supplementary Information

Supplementary Material 1.

Supplementary Material 2.

Supplementary Material 3.

Supplementary Material 4.

## Data Availability

The datasets generated and analyzed during the current study are available from the corresponding author on reasonable request. All RNA sequencing datasets were deposited in the National Center for Biotechnology Information’s Gene Expression Omnibus with accession number GSE300993.

## Abbreviations

OA: Osteoarthritis
SA-β-gal: Senescence-associated beta-galactosidase
SASP: Senescence-associated secretory phenotype
SFs: Synovial fibroblasts
3D: Three-dimensional
PBS: Phosphate-buffered saline
α-MEM: α-Modified minimum essential medium
FBS: Fetal bovine serum
H_2_O_2_: Hydrogen peroxide
HE: Hematoxylin and eosin
TBS-T: Tris-buffered saline containing 0.1% Tween-20
DEGs: Differentially expressed genes
MMT: Medial meniscus transection
GO: Gene Ontology
KEGG: Kyoto Encyclopedia of Genes and Genomes
GSEA: Gene Set Enrichment Analysis
ECM: Extracellular matrix
PPI: Protein-protein interaction
UMAP: Uniform manifold approximation and projection
