# Supplementary material for: Generation of a senescent synovial organoid model using rat synovial fibroblasts treated with oxidative stress
Source: Inflamm Regen. 2026 Aug 5;46:41. doi: 10.1186/s41232-026-00436-2 (PMC13725996; doi:10.1186/s41232-026-00436-2)
Supplement: Supplementary file 1 [file 41232_2026_436_MOESM1_ESM.docx]

**Supplementary Fig. 1** Transcriptome characterization of SFs growing as monolayers. (a) Heatmap of differentially expressed genes between Cont-ML and Sen-ML. (b) Gene ontology analysis of genes upregulated in Sen-ML versus Cont-ML. The top 10 terms are shown as a bubble plot. (c) Kyoto Encyclopedia of Genes and Genomes pathway analysis of genes upregulated in Sen-ML versus Cont-ML. The top 10 terms are shown as a bar chart. Cont-ML: untreated control SFs cultured in monolayers; Sen-ML: senescent H_2_O_2_-treated SFs cultured in monolayers

**Supplementary Fig. 2** Representative images of immunostaining for Prg4 and Cd90 in control organoids and senescent organoids. Cont-Org: untreated control SFs cultured as organoids; Sen-Org: senescent H_2_O_2_-treated SFs cultured as organoids

**Supplementary Fig. 3** Venn diagram of differentially expressed genes in Cont-Org versus Cont-ML, Sen-Org versus Sen-ML, Sen-ML versus Cont-ML, and Sen-Org versus Cont-Org. Cont-ML: untreated control SFs cultured in monolayers; Sen-ML: senescent H_2_O_2_-treated SFs cultured in monolayers; Cont-Org: untreated control SFs cultured as organoids; Sen-Org: senescent H_2_O_2_-treated SFs cultured as organoids

**Supplementary Fig. 4** Expression levels of Il1a, Cdkn1a, Cdkn2a, Rcan1 and Angptl4 in organoids. Data were expressed as transcripts per million (TPM). Cont-Org: untreated control SFs cultured as organoids; Sen-Org: senescent H_2_O_2_-treated SFs cultured as organoids

**Supplementary Fig. 5** Single-cell RNA sequencing of human healthy and OA synovium. (a) Uniform manifold approximation and projection plot after cell type annotation. (b) Bubble plot of marker genes for each cell type.

**Supplementary Fig. 6** Protein‒protein interaction analysis of genes upregulated in OA vs. healthy in the sublining (a), lining (b), and intermediate SF subset (c). Genes with 20 or more interactions are visualized using Cytoscape. The size of each node is proportional to the number of interactions.
